# Peak MSC—Are We There Yet?

**DOI:** 10.3389/fmed.2018.00178

**Published:** 2018-06-21

**Authors:** Timothy R. Olsen, Kelvin S. Ng, Lye T. Lock, Tabassum Ahsan, Jon A. Rowley

**Affiliations:** RoosterBio, Inc. Frederick, MD, United States

**Keywords:** mesenchymal stem cell, regenerative medicine, stem cell, cell therapy, cell manufacturing, bioprocessing

## Abstract

Human mesenchymal stem cells (hMSCs) are a critical raw material for many regenerative medicine products, including cell-based therapies, engineered tissues, or combination products, and are on the brink of radically changing how the world of medicine operates. Their unique characteristics, potential to treat many indications, and established safety profile in more than 800 clinical trials have contributed to their current consumption and will only fuel future demand. Given the large target patient populations with typical dose sizes of 10's to 100's of millions of cells per patient, and engineered tissues being constructed with 100's of millions to billions of cells, an unprecedented demand has been created for hMSCs. The fulfillment of this demand faces an uphill challenge in the limited availability of large quantities of pharmaceutical grade hMSCs for the industry—fueling the need for parallel rapid advancements in the biomanufacturing of this living critical raw material. Simply put, hMSCs are no different than technologies like transistors, as they are a highly technical and modular product that requires stringent control over manufacturing that can allow for high quality and consistent performance. As hMSC manufacturing processes are optimized, it predicts a future time of abundance for hMSCs, where scientists and researchers around the world will have access to a consistent and readily available supply of high quality, standardized, and economical pharmaceutical grade product to buy off the shelf for their applications and drive product development—this is “Peak MSC.”

## Introduction

The global regenerative medicine (RegenMed) industry has seen tremendous progress over the last decade with advancements in cell therapy, biofabrication, and synthetic biology—all of which are presenting life-saving treatment options for patients with unmet medical needs. Human mesenchymal stem cells (hMSCs) are a critical raw material for many RegenMed products, whether they are cell-based therapies, engineered tissues, or combination products. Given the large target patient populations with typical dose sizes of 10's to 100's of millions of cells per patient, and engineered tissues being constructed with 100's of millions of cells, an unprecedented demand has been created for these cells (1, 2). This demand faces an uphill challenge in the limited availability of large quantities of pharmaceutical grade MSCs for the industry—fueling the need for parallel rapid advancements in the biomanufacturing of these unique cells.

hMSCs are more than simply tools for basic research; they are more akin to a technology like a transistor in a microchip. Industry leaders, like Bill Maris, President & Managing Partner of Google Ventures, has stated that biology is having its' “transistor moment” due to RegenMed technologies like stem cells, where their therapeutic applicability is increasing exponentially, drawing parallels to computing (3). The demand for hMSCs already exists with current clinical applications. As of December 2017, www.clinicaltrials.gov listed 837 registered clinical trials involving hMSCs (with 102 added in 2017 alone) targeting a host of indications, ranging from graft vs. host disease (GvHD) to macular degeneration to osteoarthritis. Since many of the trials are in early to mid-stage clinical development and commercial products are just beginning to receive marketing approval around the world, hMSC demand will continue to increase for years to come. Given their widespread therapeutic potential for treating many indications and usage in a variety of applications, the authors believe that hMSCs are the “microchips of tomorrow's RegenMed products.”

hMSCs have been used as a biological tool to understand cellular mechanisms, prior to their growth into development and therapeutic applications in RegenMed. The change in types of research using hMSCs has triggered the demand for the cells in large quantities and pharmaceutical grade. For example, a typical academic publication for *in vitro* cell therapy consumes about 43M hMSCs, while an average clinical trial will command 60B hMSCs to treat each patient, as calculated by the authors and discussed in detail later in the manuscript. Recently, gains have been made to address this demand due to a focus on manufacturing process sciences and the RegenMed field is on the brink of manufacturing breakthroughs to propel hMSCs to become an abundant resource available to all researchers. Peter Diamandis described a set of “laws of abundance” in his 2012 book “Abundance: the Future is Better than you Think” to explain that technology is a “resource-liberating force (4).” The authors envision that the laws of abundance that drive mass adoption of technology products will also apply to the state of MSCs in commercial therapeutic applications. One can think of this type of demand being derived from increased supply—or abundance—of hMSCs. Rob Carlson, the authoritative tracker of progress in biotechnology over time, stresses that “biology is technology” and in his book describes, “New technologies provide opportunities to expand markets or launch entirely new ones (5).” His insight falls right in tune with the future potential of hMSCs. That is; if manufacturers can develop new technologies and processes that deliver high quality hMSCs in massive volumes and at radically lower costs, then their use will skyrocket in existing and emerging markets. This concept follows the path that led us from having a single telephone in a building just a few years ago, to today where every single occupant has a personal cell phone (which doubles as a high-powered computer) in their pocket.

While hMSCs hold tremendous promise due to a strong signal of therapeutic efficacy in early stage trials and a growing number of later stage clinical trials, this has also come with disappointment. A high-level snapshot of the MSC industry is captured in aftermath of California Proposition 71. In 2004, the California Institute for Regenerative Medicine was born thanks to a $3 Billion investment provided by the United States government after voters approved California Proposition 71 (6). Over the next 10 years, 750 grants were distributed, a dozen research facilities were constructed, nearly 2,000 papers were published, more than 2,400 students and scientists were trained, and 30 projects (including clinical trials) were funded (6). After all of this—CIRM has not generated a therapy approved for commercial use. Having no tangible commercialized product is of concern, but this “gap” to commercialization can be attributed to the reality of how long the “drug” development timeline is, with most drugs taking at least a decade and over a billion dollars before reaching market. There was further disappointment in a failed set of late-stage clinical trials. In 2008/9, Osiris reported the failure of multiple Phase 3 trials using a stem cell product called Prochymal for the treatment of GvHD, with no difference compared to placebo, with other mid-stage trial disappointments within the last 5–7 years.

While there have been setbacks for the cell therapy industry and no FDA approvals for stem cell based products in the USA, there is a silver lining in that there are approved stem cell based products on the global market, including Prochymal (Osiris, approved in Canada), Alofisal (TiGenix and Takeda, approved in Europe), Temcell (JCR Pharmaceuticals, approved in Japan), HeartSheet (Terumo, approved in Japan), Cartistem (Medipost, approved in South Korea), and Hearticellgram-AMI (FCB-Pharmicell, approved in South Korea). In February 2018, Mesoblast announced the success of a Phase 3 GvHD trial, demonstrating a 70% response rate with MSC-100-IV treatment, compared to the historical control rate of 45%. This efficacy data, paired with 180-day safety and quality of life data is expected to provide the means for accelerated FDA approval. Another highlight can be found in the FDA's granting of 10 Regenerative Medicine Advanced Therapy Designation requests in 2017, which provides expedited review for therapies targeting serious or life-threatening diseases, given that there is preliminary clinical evidence that the therapy can treat otherwise unmet medical needs. Taken together, the above approved products, a success in a late phase clinical trial, and the FDA working to accelerate promising cell therapies, portray a bright future for stem cell based therapies.

The field is still directing efforts on how to manufacture consistent product at scale, deliver the product to the patient in a way that maximizes therapeutic efficacy, as well as how to design and execute successful clinical trials—none of these are trivial challenges! These challenges could potentially represent a roadblock to commercial success, resulting in constraining the future demand and number of applications using hMSCs. The authors, in this manuscript, conjecture that with increasing government investment in cell manufacturing, the vast number of indications where hMSCs show a strong therapeutic signal, and the increasing availability of high volume, high quality and low cost hMSCs on the market, the peak demand for these cells is still far into the future.

In this manuscript, we utilize historical data to understand how hMSC demand has grown over time to today's volumes, and what applications drive this growing demand. We also highlight current and future applications utilizing hMSCs and propose what future hMSC demand will look like based on data-supported assumptions related to indications, doses, and conservative market adoption scenarios. We discuss potential solutions to manufacturing challenges that will be necessary to meet this ever-growing demand, and we also suggest what technologies will eventually disrupt hMSCs, and ultimately predict a “peak” hMSC demand.

## Historic MSC consumption

### Academic and preclinical

**Figure 1:**
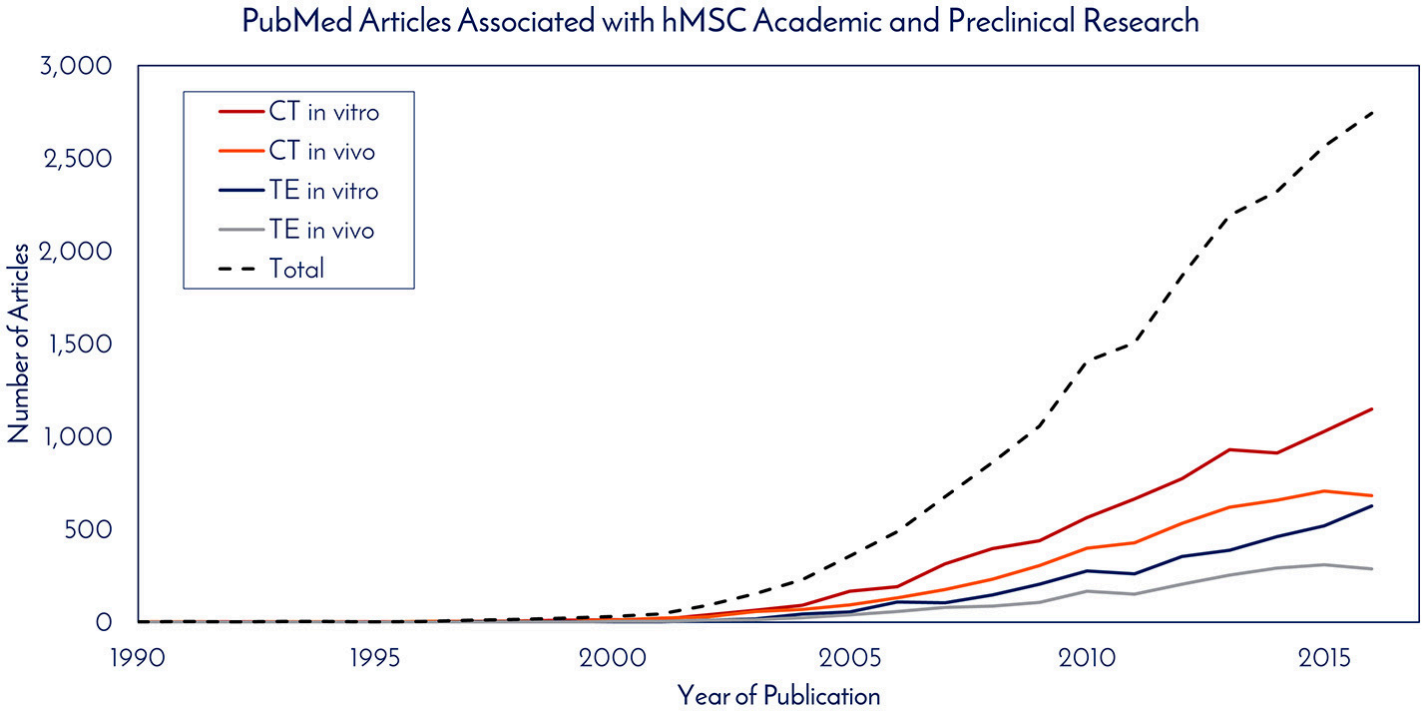
PubMed articles associated with hMSC academic and preclinical research. The number of articles published each year that utilized hMSCs for *in vitro* and *in vivo* work in cell therapy (CE) and tissue engineering (TE) fields were totaled.

**Figure 2:**
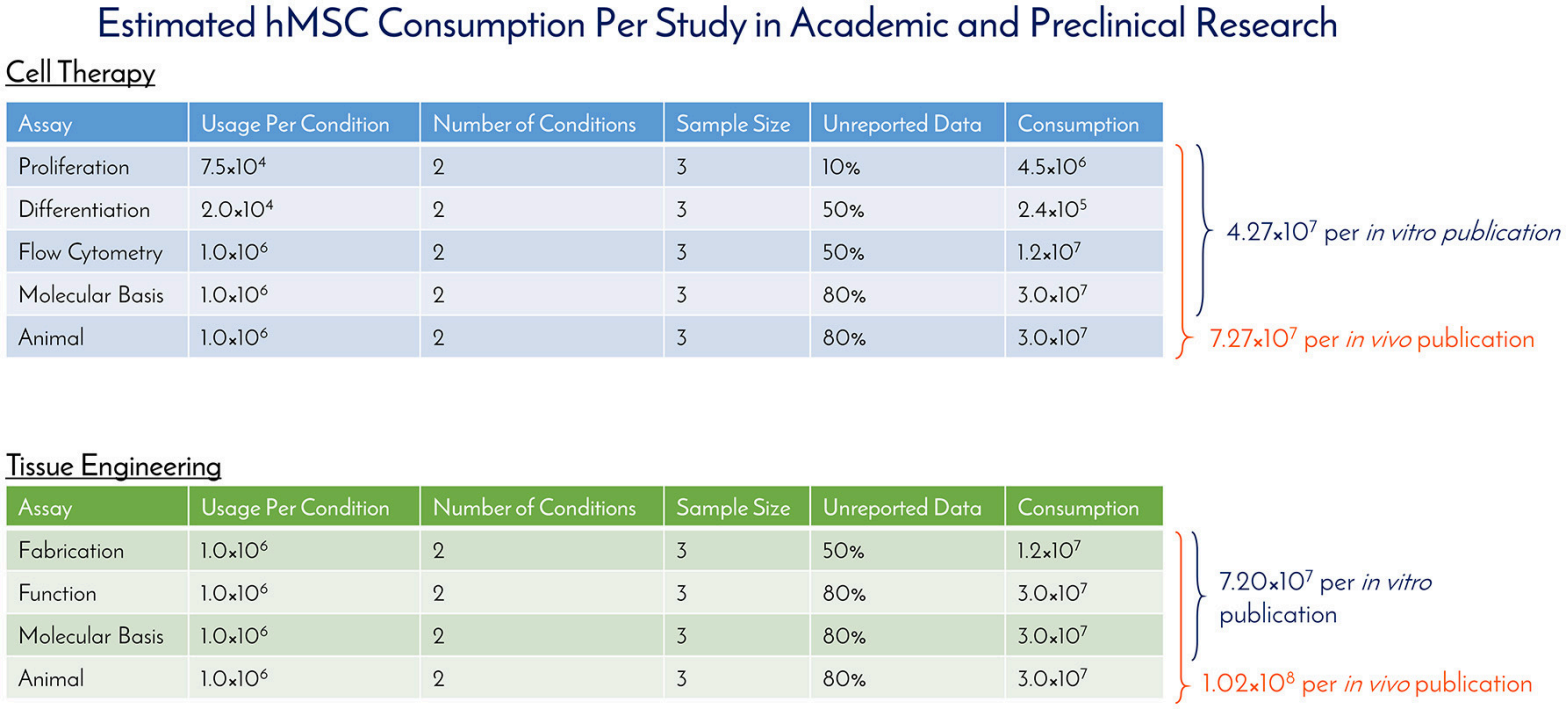
Estimated hMSC consumption per study in academic and preclinical research. The number of hMSCs consumed per *in vitro* and *in vivo* experiment were determined and used to calculate the amount of hMSCs consumed per publication for cell therapy and tissue engineering fields.

**Figure 3:**
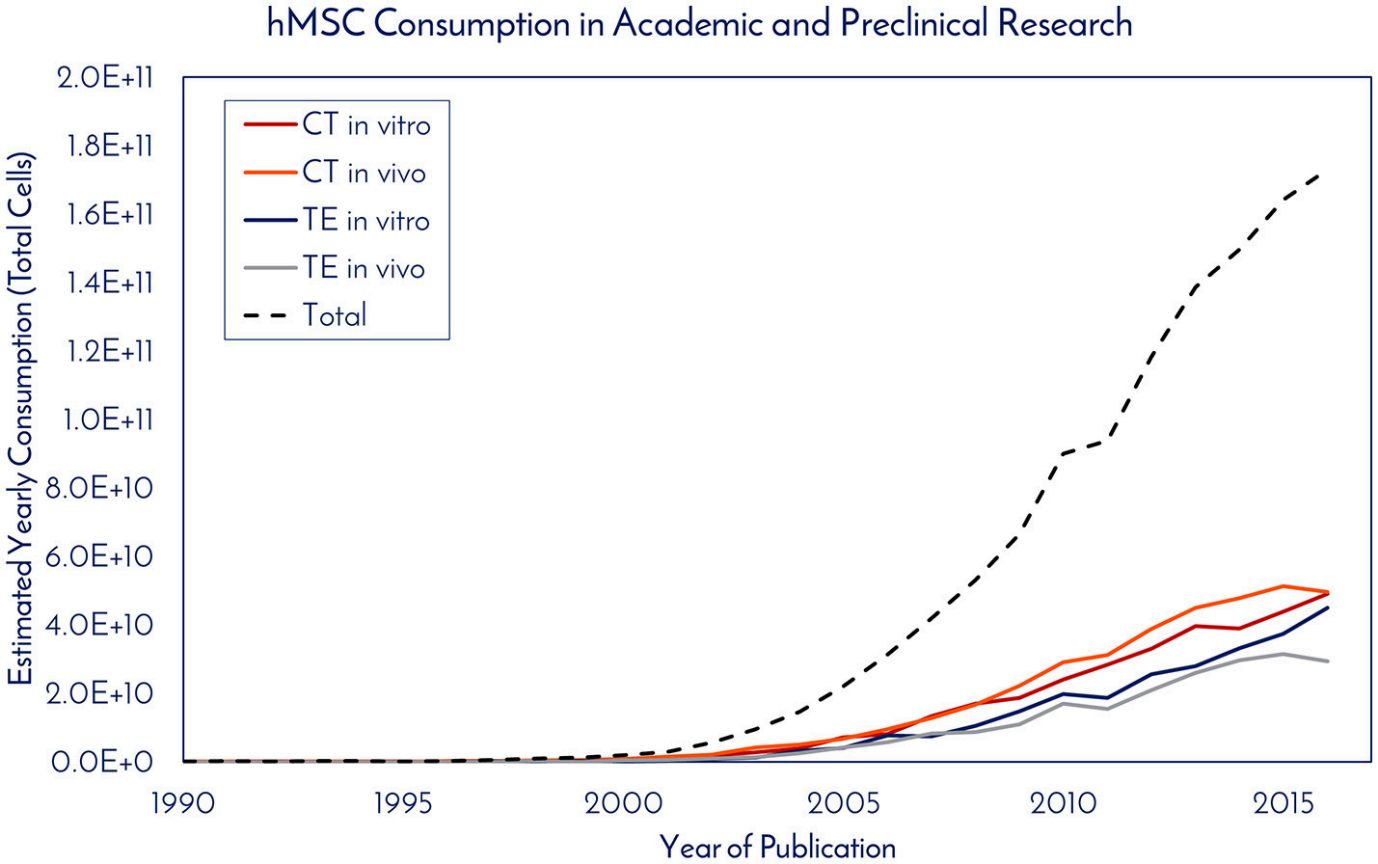
hMSC consumption in academic and preclinical research. The estimated yearly consumption of hMSCs for *in vitro* and *in vivo* work in publications from the cell therapy (CE) and tissue engineering (TE) fields was calculated based on the number of articles published per year and the amount of hMSCs consumed to generate the data required for publication.

Historically speaking, hMSCs have been in the literature since before Arnold Caplan coined the term “mesenchymal stem cell” in the late 1980s, with early work published by Friedenstein and collaborators in the 1960s that didn't use the MSC nomenclature (7). For studying historical consumption of hMSCs in the literature, 1990 was used as the start date (the year of birth of the youngest author on this paper) and through 2016 as the end date. Supplemental Figure 1 details the selection criteria on PubMed for determining the number of publications using hMSCs for tissue engineering (TE) and cell therapy (CT), while separating *in vitro* and *in vivo* usage. The respective numbers of PubMed articles associated with hMSC academic and preclinical research over time are graphically depicted in Figure 1. Of the 2,744 primary research articles published in 2016, CT *in vitro*, CT *in vivo*, TE *in vitro*, and TE *in vivo* accounted for 42, 25, 23, and 10% of the total, respectively. The total number of articles published in 2016 represents a 770% increase from the articles published in 2005. To ascertain the total number of cells used per publication, a list of the most common experiments was constructed for CT and TE publications (Figure 2). Based on the publication records and previous experience of the authors, we created assumptions for the number of cells per data point, number of conditions tested, sample size, unreported data, and total consumption per assay were calculated. Unreported data represents the assumption that not all experiments performed in support of the publication made it to the final print. For CT, *in vitro* and *in vivo* experiments consumed 4.27 × 10^7^ and 7.27 × 10^7^ hMSCs per publication, respectively. For TE, *in vitro* and *in vivo* experiments consumed 7.20 × 10^7^ and 1.02 × 10^8^ hMSCs per publication, respectively. Based on these calculations, the total estimated yearly consumption of MSCs in academic and preclinical research was graphed over time (Figure 3). In 2016, it is estimated that 1.73 × 10^11^ hMSCs were consumed in academic and preclinical research. Of this total, CT *in vitro*, CT *in vivo*, TE *in vitro*, and TE *in vivo* accounted for 28, 29, 26, and 17% of the total cell consumption, respectively.

### Clinical

**Figure 4:**
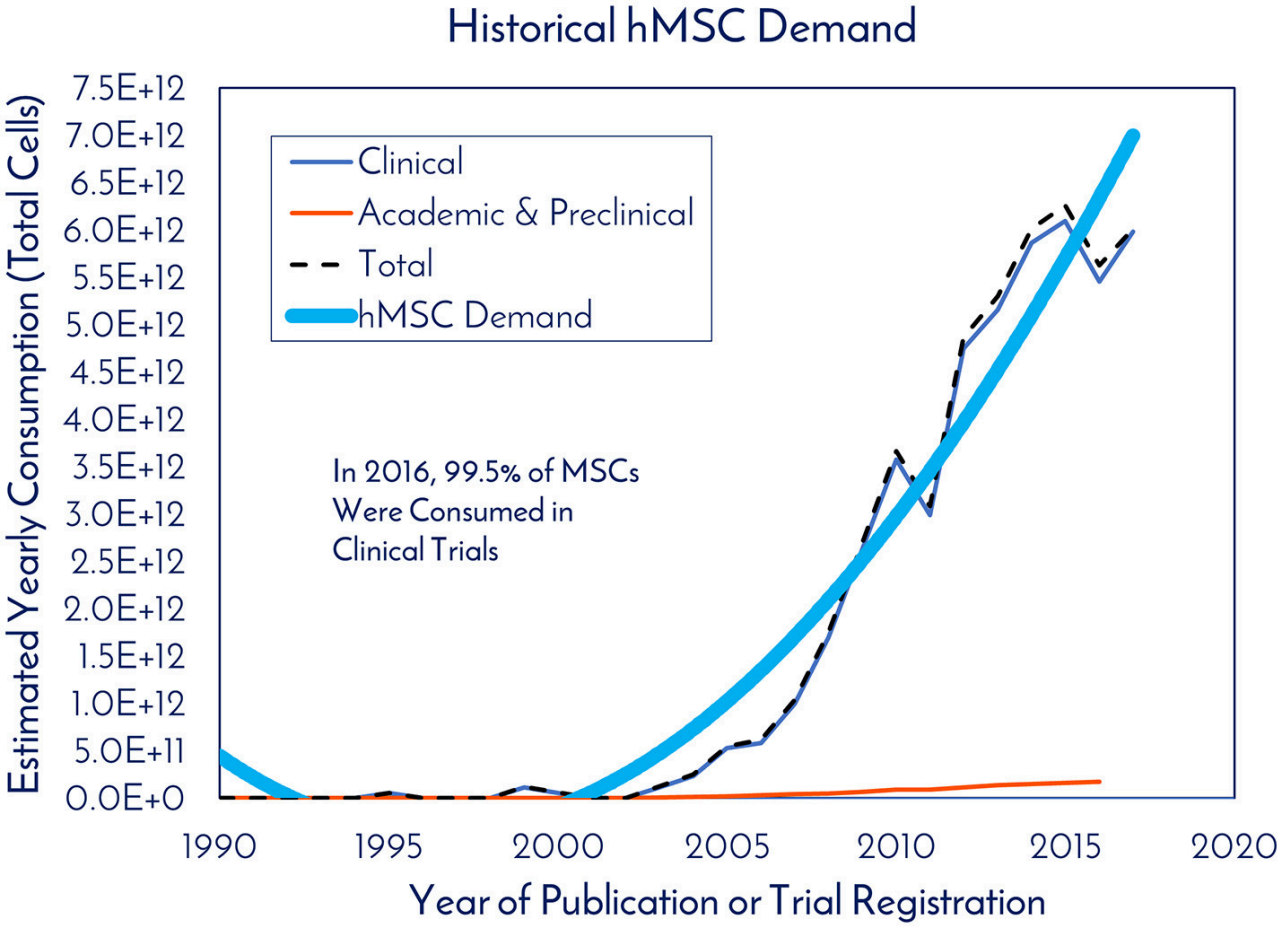
Historical hMSC demand. The estimated yearly consumption of hMSCs from Clinical and Academic/Preclinical work. Clinical consumption was determined based on reported cell consumption from registered clinical trials and the total amount of registered trials each year (clinicaltrials.gov).

For studying historical consumption of hMSCs in clinical trials, dosage information and patient totals were extracted from the clinical trials that reported it. At the time of the search in November 2017, of the 814 registered clinical trials, only 299 trials reported both pieces of data, which we then used to calculate the total cells consumed per clinical trial (Supplemental Figure 2). Based on these trials, there was an average of 47 patients per trial, 416M cells used per patient, and 19.4B cells consumed per clinical trial (Supplemental Figure 2). With a 3x manufacturing safety factor incorporated to ensure enough potential follow up doses are available, we estimate that 60B cells would need to be manufactured per clinical trial. After determining the number of cells used per trial, this number was multiplied by the total number of trials in a given year, yielding what we called “Estimated Yearly Consumption” in clinical trials (Figure 4). Here, academic and preclinical consumption was included on the graph with clinical consumption to highlight the difference. In 2016, 99.5% of hMSCs were consumed in clinical trials and 0.5% of hMSCs were consumed in academic and preclinical work. The total number of hMSCs consumed in clinical trials in 2017 represents a 1,133% increase from the hMSCs consumed in clinical trials in 2005.

**Figure 5:**
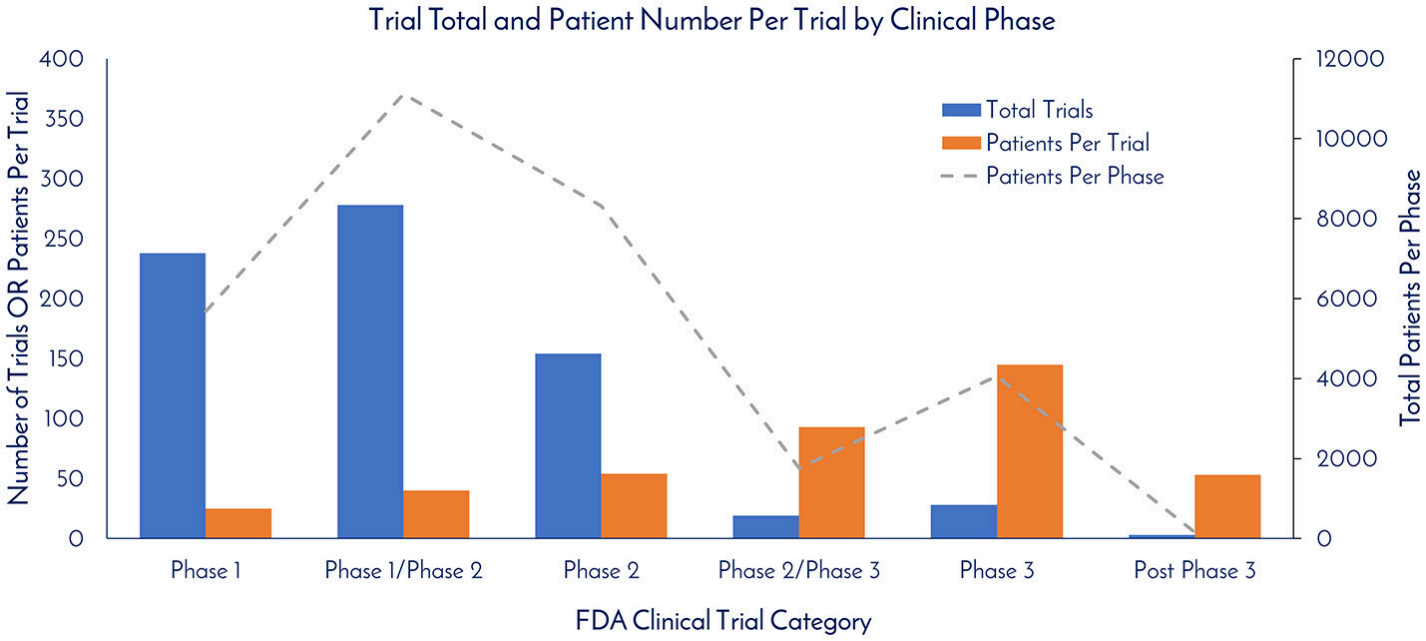
Trial total and patient number per trial by clinical phase. The total trials per clinical phase, the average patient number per trial per phase, and the total amount of patients per clinical phase were calculated based on currently registered clinical trials (clinicaltrials.gov).

As of December 31, 2017, there were 837 registered clinical trials. Of this total, 720 of the trials reported the Phase. There were 238, 278, 154, 19, 28, and 3 registered trials in Phase 1, Phase 1/Phase 2, Phase 2, Phase 2/Phase 3, Phase 3, and Post-Phase 3 stages, respectively (Figure 5). The patients per trial for each phase was calculated by taking the total patients in each phase and dividing by the total trials in each respective phase. There were 25, 40, 54, 93, 145, and 53 patients per trial in Phase 1, Phase 1/Phase 2, Phase 2, Phase 2/Phase 3, Phase 3, and Post-Phase 3 stages, respectively (Figure 5).

hMSCs have been identified for use in a long list of therapeutic applications (clinicaltrials.gov). A snapshot of hMSC target applications with indication, total trials per indication, and cells used per patient per indication is listed in Table 1. Bone and Cartilage, Cardiovascular Disease, and Diabetes are the top three indications in terms of relative percent of total trials, with 11.22, 15.29, and 6.41% of the total, respectively. Crohn's Disease, GvHD, and Lung Disease had the most cells used per patient, with 1.508B, 578M, and 451M, respectively.

**Table 1:** hMSC target indication trial numbers and cells per patient.

**Indication**	**Total trials**	**Percent of trials (%)**	**Cells per patient (M cells)**
Alzheimer's disease	10	1.23	174
Amyotrophic lateral sclerosis	19	2.34	126
Bone and cartilage	91	11.22	101
Cancer	39	4.81	132
Cardiovascular disease	124	15.29	120
Crohn's disease	26	3.21	1,508
Diabetes	52	6.41	375
Erectile dysfunction	10	1.23	15
GvHD	42	5.18	578
Hematological disease	28	3.45	192
Kidney disease	35	4.32	261
Liver disease	50	6.17	420
Lung disease	47	5.80	451
Lupus	8	0.99	70
Mutliple sclerosis	24	2.96	190
Other	208	25.40	N/A
Parkinson's disease	3	0.37	168
Psoriasis	3	0.37	420
Spinal cord injury	49	6.04	109

## Applications driving growth of future MSC consumption through 2040

### MSC-therapeutic products (MSC-TP)

**Figure 6:**
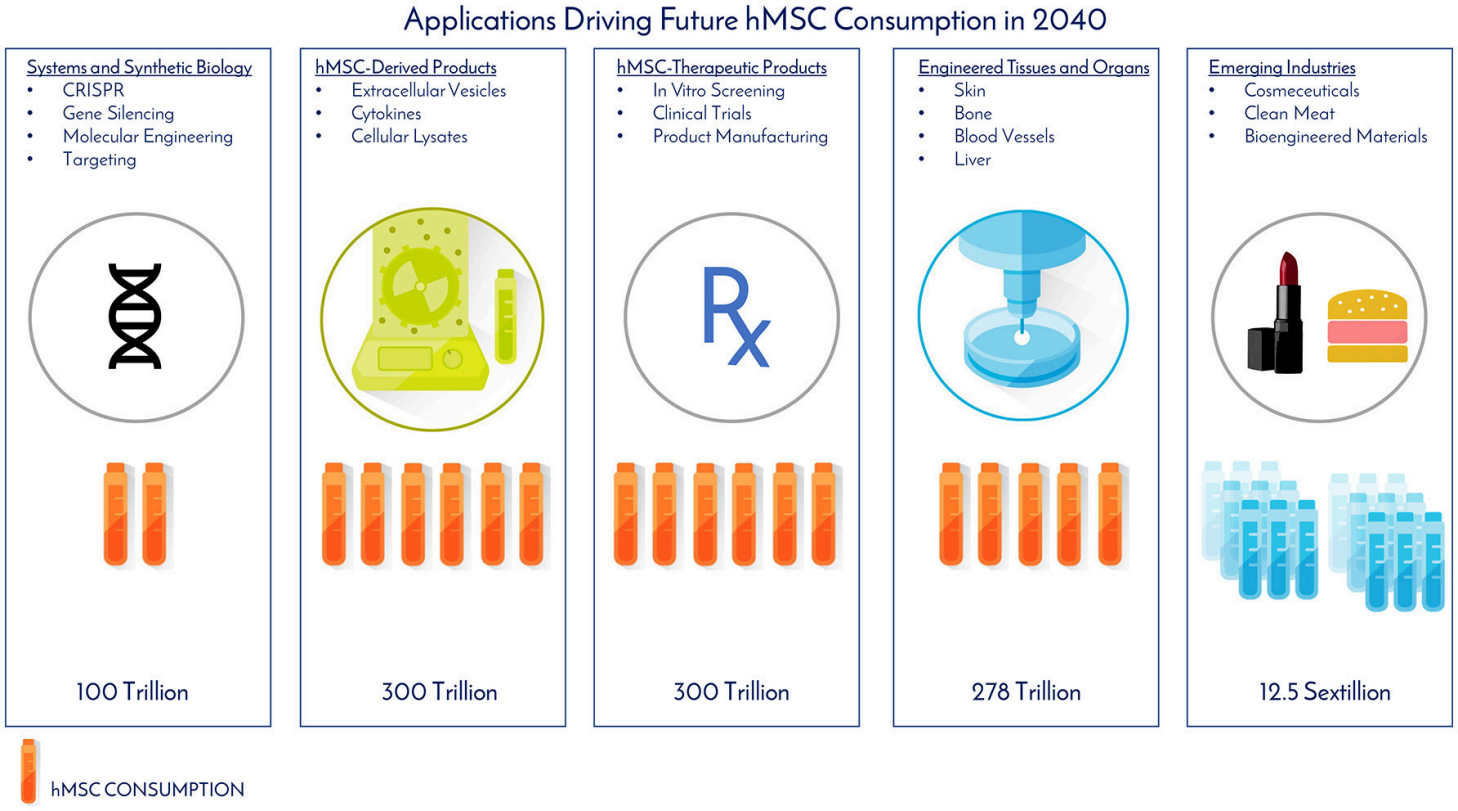
Applications driving future hMSC consumption in 2040. The projected total number of hMSCs consumed per application and industry was determined by assumptions from historical clinical data, current consumption rates, and future projections based on the experiences and expertise of the authors.

**Figure 7:**
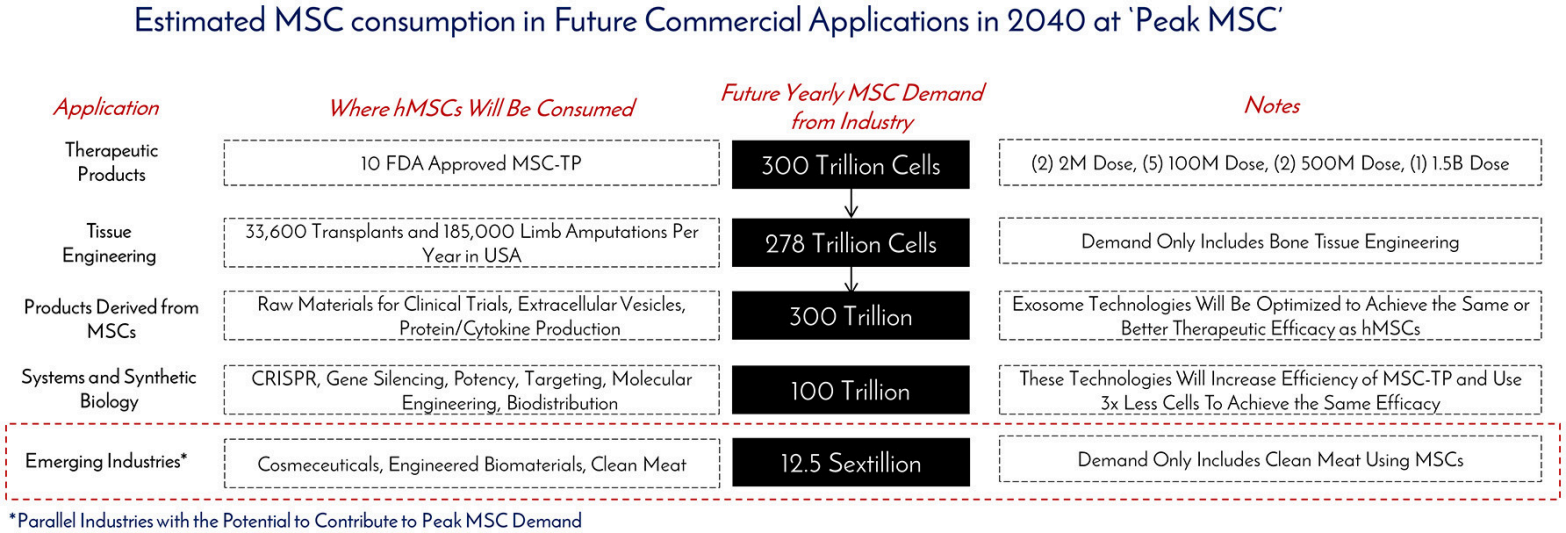
Estimated MSC consumption in future commercial applications in 2040 at “Peak MSC.” The different types of applications requiring MSCs, how they will be consumed, and the total amounts of cells to be consumed were determined based on historical, current, and projected usage of MSCs as manufacturing technologies create an abundance of this critical raw material at “Peak MSC” in 2040.

As of November 2017, there were 46 Phase 2/Phase 3 and Phase 3 clinical trials using hMSCs and the FDA estimates that 25–30% of Phase III clinical drugs receive approval (8). Our conservative prediction calls for 10 MSC-TP to gain FDA approval for sale on the market by 2030. The target applications and areas of consumption for these MSC-TPs will be clinical trials, commercial product sales, and *in vitro* screening (Figure 6). One promising application of hMSCs is for the treatment of GvHD. Each year, 25,000 people receive hematopoietic stem cell transplants, with acute and chronic GvHD developing in 25–80% of the patients, depending on human leukocyte antigen (HLA) matching, creating a yearly patient population of about 12,500 that would need treatment with the hMSC drug (9). Based on clinical trials using hMSCs for treating GvHD, the average patient requires 578M cells. We estimate that Manufacturers will need to generate a minimum of 2x more doses than what are required for patients, calling for 25,000 doses to be generated per year. This translates to a demand of 14.5 trillion hMSCs for the treatment of GvHD per year. GvHD has a small patient population when compared to diabetes, with 12,500 and 1,500,000 new patients diagnosed each year, respectively (10). The average patient in a clinical trial, regardless of indication, requires 416M cells. When 10 MSC-TP reach market, we assumed there will be 2 “Low Dose” Products with 2M cells/dose, 5 “Medium Dose” Products with 100M cells/dose, 2 “High Dose” Products with 500M cells/dose, and 1 “Monster Dose” with 1.5B cells/dose. Given the wide range of potential patient populations, a flat 100,000 patients per indication is assumed. Based on these assumptions, 300 Trillion hMSCs would be required per year to satisfy this demand (Figures 6, 7). If MSC-TPs are approved for high patient population indications, like diabetes or stroke, this forecast could easily multiply by 10-fold.

### Engineered tissues and organs

In the United States, there are more than 116,000 people currently waiting for lifesaving organ transplants (kidney, liver, heart) and 185,000 upper and lower limb amputations performed each year (11, 12). Tissue engineering, especially biofabrication, offers new hope and therapeutic options for these patients that were otherwise left with a poorer quality of life. hMSCs have been established as one of the ideal cellular starting material for biofabrication of living constructs due to decades of data on their safety in clinical trials and ability to treat a variety of injuries and diseases ranging from regeneration of bone and cartilage, to the treatment of stroke and cancer. Bioprinting is a rapidly evolving tissue engineering segment that holds a lot of promise for product customization, and to address the worldwide tissue and organ shortage, with its global market expected to reach $1.82 Billion USD by 2022 (13).

While bioprinting has emerged as a promising technology for additive manufacturing, a challenge exists in generating enough cellular starting material to manufacture tissues like bone and guiding the printed structure to behave like a bone—or as Jordan Miller expressed, “will function follow form Miller (14)?” In John Fisher's lab, a bioprinted bone scaffold the size of the superior half of an adult human femur was seeded with 720M hMSCs encapsulated in alginate beads and subjected to dynamic culture in a bioreactor (2). This construct was 20-fold larger in size than any tissue engineered bone construct reported to date, and also achieved high cell viability through the graft with early signs of stem cell differentiation through increased expression of osteogenic genes and markers (2). This work gives good insight into what the future hMSC demand will be as this technology moves from benchtop to bedside. Assuming 185,000 upper and lower limb amputations are performed per year, this prompts a demand for 185,000 tissue engineered bones to replace those that were lost. From Fisher's study, the superior portion of the femur was engineered with 720M hMSCs, meaning a full femur would require about 1.5B hMSCs. This number was used for forecasting purposes. To fulfill the demand for the all the patients requiring replacement bones, 278 trillion cells would be needed per year (Figures 6, 7). The predicted hMSC consumption for bone tissue engineering was only used for forecasting tissue engineering consumption to be conservative.

Tissue engineering of organs has its own set of challenges—generating the cell numbers required for manufacturing, mimicking the complex structural organization, creating a vascular network, and achieving sustained functionality, just to name a few (14). Creating a functional tissue engineered liver is one of the “holy grails” of bioprinting as this feat would be an engineering marvel and change the medical world forever. Out of the 33,611 transplants performed in 2016, 7,841 were livers. Furthermore, out of the 116,000+ patients currently waiting for transplants in 2016, 6,427 people were waiting for a liver, meaning half of the patients are still waiting. Only half of a human liver is required to support life and is suitable for transplant, so this was used for forecasting purposes (15). A healthy human liver has been estimated to contain 360 billion cells, but research suggests that acute liver failure patients can be supported with 5–10% liver mass or bioartificial liver composed of 10 billion cells (16, 17). While hepatocytes are the functional cell of the liver, stem cells have been recently shown to play a functional role in liver maintenance and regeneration, in addition to being a non-parenchymal cell precursor (17, 18). hMSCs have also been shown to have the ability to be directed toward differentiation into hepatocytes (19). Hepatocytes (parenchymal cells) make up 60% of the liver cell total, while non-parenchymal cells (endothelial cells, Kupffer cells, and stellate cells) make up the remaining 40% (20). On a conservative estimate, it is assumed 25% of the non-parenchymal cells will be hMSCs in a tissue engineered liver, hence 1B hMSCs in total. To bioprint enough livers to meet the demand for the patients left waiting at the end of 2016, 6,427 livers would need to be manufactured, which translates to 6.427 Trillion hMSCs (11). The Department of Health and Human Services shows that livers represent just 12.3% of the organs transplanted in 2016, which foreshadows a huge demand for hMSCs as tissue engineering of organs develops (11). Assuming hMSCs can account for generating 25% of non-parenchymal cells in solid organs (kidney, liver, pancreas) and that non-parenchymal cells make up 40% of the solid organs, an additional 10-fold multiplication factor would be needed to satisfy all organ manufacturing and to begin long term organ storage of off-the-shelf-use, totaling 64 trillion cells. The hMSC demand for these applications is of course dependent on cell production, differentiation and maturation technologies of the parenchymal cells needed for organ function; which is a topic that will be more appropriately addressed by researchers in the field. However, we believe that the availability of abundant hMSC source through manufacturing technology innovations will lay the groundwork for rapid progress in those areas as well.

### MSC-derived products

Limited *in vivo* persistence of transplanted hMSCs despite long-term therapeutic benefits has strongly implicated secretory factors as the dominant mode of hMSC action, of which extracellular vesicles (EVs) such as exosomes are particularly potent (21–23). As complex vehicles of biological signals, EVs can elicit therapeutic responses comparable to their cellular counterparts, demonstrating a capacity for MSC-EVs to become a cell-free alternative to MSC therapy (24). Meanwhile, a rapidly emerging industry is harnessing the natural signaling ability of EVs to deliver exogenous agents including RNA and proteins (25). Given their history of clinical use without significant adverse events, hMSCs are a clinically relevant cell source valued for its potential in accelerating translation of EV therapies (26, 27).

EVs may be manufactured on their own, or in parallel with cell therapies, and scalable EV bioprocesses are on the horizon (28). However, based on the current state of the art, the MSC-EV dose required to achieve the same therapeutic efficacy of hMSCs has not been optimized in large scale human studies (29, 30). One registered clinical trial (clinicaltrial.gov #NCT02138331) using MSC-EVs is in progress for the treatment of Type 1 Diabetes with a dose range of 1.22–1.51 × 10^6^/kg, but no information is given on the cells required to generate this dose. One in-man study with a single patient utilized 40M MSCs (corresponding cell dose for an hMSC treatment) to generate a “unit” dose of MSC-EV for the treatment of GvHD. Seven doses with increasing amounts of exosomes per dose were delivered over 2 weeks with no side effects. The patient responded within days of treatment and stabilized after 4 months. Eight units in total were used for one patient, translating to 320M hMSCs per patient. Compared to the clinical data, multiple cell doses, up to a total of 578M hMSCs can be used per patient in GvHD clinical trials (Table 1). Though only one data point is available, we assume MSC-Derived Products, like MSC-EVs, will require an equivalent, if not higher amount, of hMSCs for treatment at the moment. Based on this, we assume the same demand as MSC-Therapeutic Products (300 Trillion) over the next 20 years (Figure 7). Again this is on the conservative side, as this does not include MSC-derived materials, like cytokines and cell lysates, both of which have shown therapeutic promise (31–33) (Figure 6). Toward clinical-scale manufacturing, MSC-derived applications will create a tremendous demand for MSCs.

### Emerging industries

In addition to cell therapy, hMSCs are poised to transform other industries and markets (Figure 6). Cosmeceuticals will represent a $61 Billion market by 2020, and companies like L'Oreal and Johnson and Johnson are directing resources toward giving makeups and personal care products a biological component, targeting growth factors and cytokines from MSCs as potential additives (34). Even more, these companies are aligning themselves toward doing makeup and personal care product testing on bioprinted human skin models, as animal testing for final products and marketing of products that were tested on animals has been banned by the European Commission under the Cosmetics Regulation, which was established in 2013.

Another industry that has been using MSCs for development is engineered clean meat. By 2050, the global demand for meat is going to double and it is predicted each of the 10 billion people on earth will consume an average of 25–30 grams per day (35). To generate enough “meat” to “meet” this demand for a year's time, 10^23^ cells would be required (35). Assuming 3 population doublings in a typical bioreactor, this would call for 1.25 × 10^22^ cells for inoculation of all the meat bioreactors (Figure 7). Animal muscle precursor cells (satellite cells), which are derived from MSCs, are being heavily researched for this application (36). Further developments are aimed at incorporating fat cells, which can be derived from MSCs, with the muscle cells to enhance the taste of the meat product and to be more attractive and tasty for mass public consumption. In the clean meat industry, there is alignment that the current major limitation of MSC use is the high cost for generating enough cells for meeting commercially relevant scales.

### “Peak” MSC demand will occur in 2040

Early discovery of monoclonal antibody (mAb) production occurred in the 1970s, with the first mAb to be approved for human use by the FDA hitting the market in 1986 (Orthoclone OKT3) (37, 38). In 1991, Centocor was given marketing approval in Europe for their first mAb drug, Centoxin, but was rejected by the FDA in 1992, triggering panic among the industry. Since this time, improvements in development, testing, manufacturing, and regulatory policy-making have led to having 47 approved mAb drugs on the market in Europe and the US in 2014. In 2016, it was reported that 5 of the 8 top-selling blockbuster (more than $1billion in sales) drugs were mAbs. Thirty years after the first mAb approval, the current rate of mAbs being approved per year is 4. hMSCs are poised to build on the foundation set forth by mAbs and have a similar trajectory to market development and infiltration.

As of December 31, 2017, there were 837 registered clinical trials administering hMSCs in over 36,000 patients with no significant adverse events, effectively establishing a strong safety profile. Thirty one of these trials were in Phase 3 or later, setting up the stage for approvals over the next 5 years. Between the late phase clinical trials, products already receiving market approvals in other countries, and given the 10 RMAT designations granted in 2017, hMSC products are on the brink of approval by the FDA in the United States. By analyzing historical hMSC consumption, current registered clinical trials, and developing applications utilizing hMSCs, it is clear that hMSC demand is continuing to rise at a staggering rate. Similar to the development steps of mAbs, we envision a variety of approved products to be on the market over the next 20 years as the industry moves toward peak MSC demand and consumption (Table 2): MSC-TP 1.0 (First Generation Products Composed of Neat or Minimally Modified MSCs), TE Products (Tissue Engineered Products Like Bioprinted Skin and Bones), MSC-TP 2.0 (Second Generation Products Composed of MSCs Enhanced with Systems and Synthetic Biology), Combination Products (Composed of a TE Product with MSC-TP 1.0, MSC-TP 2.0, or MSC-Derived Products), MSC-Derived Products (therapeutics generated by MSCs—exosomes, cytokines, and cell lysates), Cosmeceutical Products (personal care and makeup products incorporating one of the above MSC technologies), Emerging (MSC-based products developing in parallel industries not currently in the MSC space), and induced pluripotent stem cell therapeutic products (iPSC-TP). In Table 2, the forecasted number of approved products per product type is detailed over time. After totaling the total products between all product types, 2040 is the predicted time when the industry for hMSCs will “peak” in terms of demand and consumption. This predicts 86 total products to be on the market by 2040, or about 4 products added each year over that time, which falls in alignment with the trajectory that mAb products have taken.

**Table 2:** Forecasted approved MSC product types on the market over time.

**Product type**	**2015**	**2020**	**2025**	**2030**	**2035**	2040^*^	**2050**	**2060**
MSC-TP 1.0			5	10	5	2		
TE product				2	8	12	5	2
MSC-TP 2.0				1	5	10	8	5
Combination			2	5	10	15	20	25
MSC-derived			2	5	10	8	3	
Cosmeceuticals		5	10	15	20	25	30	30
Emerging					1	2	3	4
*iPSC*			1	3	8	12	15	10
Total	0	5	20	41	67	86^*^	84	76

* *Green color represents the year of “Peak MSC” in 2040*.

Given the potential for cosmeceuticals to use EVs or cytokines from MSCs, and not the cells themselves, it seems plausible that the cosmeceutical market could achieve and bring to market the first products using hMSCs in 2020 (Table 2). By looking at the amount of Phase 3 and later clinical trials using MSCs (currently 31), the first five MSC-TP 1.0 products could be approved and on the market by 2025. At this same time, the first iPSC-TP could establish safety data through a few clinical trials and be on the market. Additionally, the first few combination and MSC-derived (MSC-EVs) products will be reaching market. The first few approvals will open the flood gates for new products to follow the same path, as the regulatory guidelines will be set. By 2030, the first few TE products will be through clinical trials and approved on the market, along with the first MSC-2.0 product incorporating synthetic biology. Cosmeceutical products incorporating MSCs will be so common that people use them regularly in makeups and face creams. MSC-TP 1.0 will have 10 products on the market with wide spread and regular usage in the clinic.

**Figure 8:**
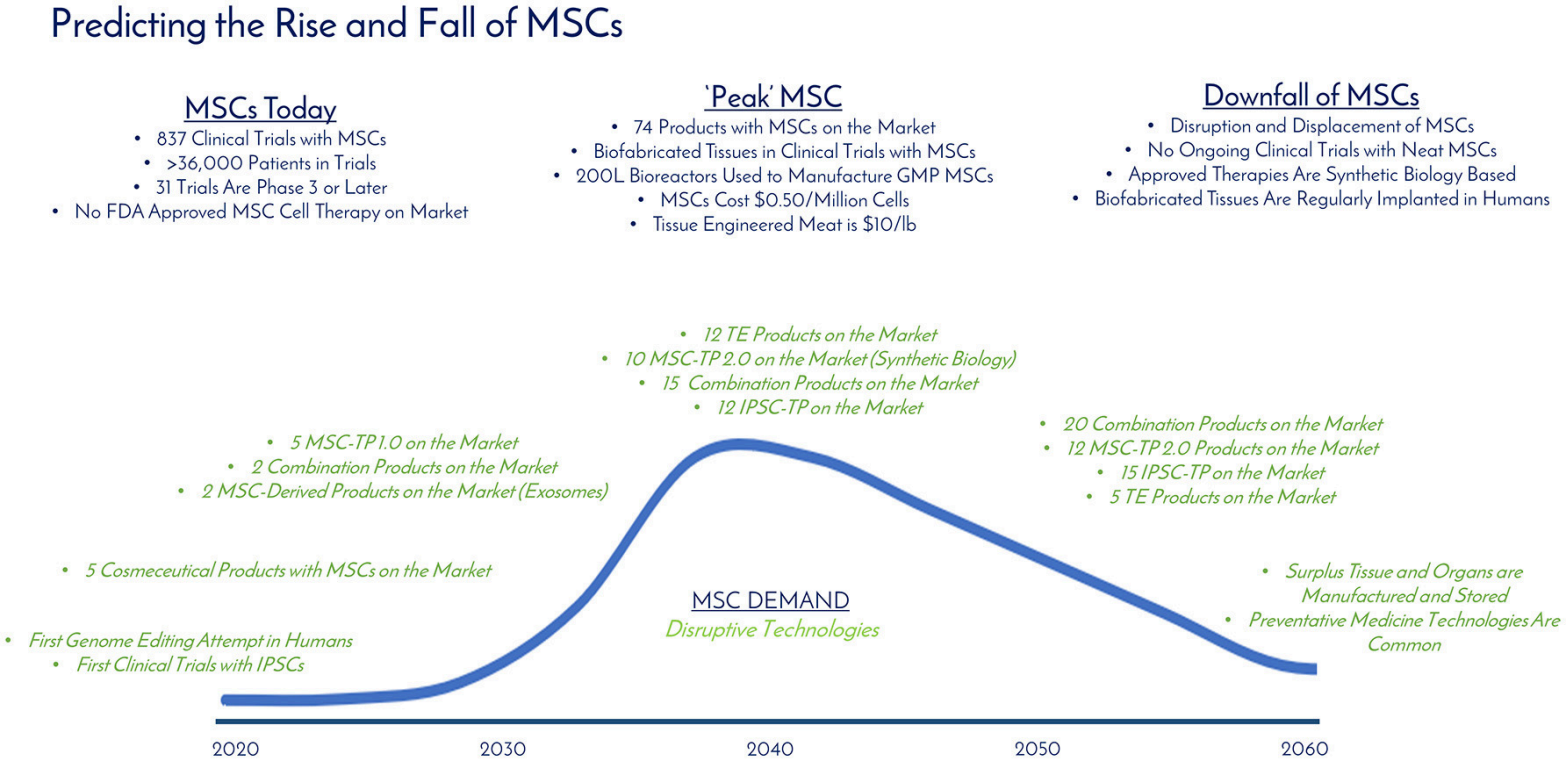
Predicting the rise and fall of MSCs. The current, “peak,” and downfall stages of MSC demand are detailed with milestones over time (blue). Disruptive technologies (green) that are developing in parallel that have the potential to displace MSC demand are detailed over time. “Peak” MSC is predicted to occur in 2040.

At “peak” MSC, we predict 74 MSC-based and 12 iPSC-based products to be on the market (Table 2). Biofabricated tissues and organs incorporating MSCs will be in clinical trials and >1000L bioreactors, or packed bed reactors, will be used to generate the cells manufactured following Good Manufacturing Practice (GMP) as the raw materials for these applications. The cost per million MSCs will be at $0.50 or below and we will be able to have engineered clean meat at the store for < $100 per pound (Figure 8). TE and combination products will dominate the market with heavily optimized and targeted therapeutic power. Large manufacturing sites will be developed and placed globally to strictly generate MSCs to meet the demand. By 2040, the abundance of this critical therapeutic material will truly be enabling innovation at the highest level. Even more, the wide-spread incorporation of hMSCs in technologies provides stability for their place in the market. Even if there is a superior technology, if hMSCs are so readily available, integrated in many clinical applications and work well, it will only make sense to keep using them.

Technology improves every day, and the authors do not believe that living cellular technology is any different—MSC demand will eventually have a peak, and then a slow downfall. Since MSCs have the potential to be integrated into so many applications (cell therapy, cosmeceuticals, clean meat, tissue engineering), it seems unlikely they will be completely eradicated, but rather enhanced by other technologies. Systems and synthetic biology is an example of a technology to enhance current MSC systems, and together will change the way medicine is approached. Combination products incorporating TE, MSCs, and synthetic biology will rule the market with 25 products addressing everything from GvHD to cancer to bioprinted bones custom made for patients in the clinic.

## MSC challenges and potential disruptions

### Technology challenges and solutions

To meet the pressing need for economic manufacturing of MSCs at clinically- and commercially- relevant scales, researchers have turned to single-use bioreactor systems that have successfully been used to manufacture other biomolecules, such as monoclonal antibodies (39). However, unlike small molecule or large molecule production, cell therapy products are living, breathing cells, which creates new bioprocessing constraints for the final product upon thaw in the clinic (39). While there are technologies based on monoclonal production that can expand MSCs in large quantities, like using 3D microcarrier based bioreactor systems, there are still many manufacturing innovations required before this manufacturing platform can support a commercial cell therapy product. The gap exists in downstream processing technologies, specifically the unit operations related to (1) harvesting the cells from the microcarriers without damaging or changing the cells in any way, (2) separating the detached cells from microcarriers in suspension using an efficient, automated methodology, and (3) concentrating large volumes of bulk cell solution in a timely manner (<5 h) to maintain cell viability and functionality.

The harvest unit operation challenge mentioned above is due to the use of proteolytic enzymes (i.e., trypsin) and agitation (i.e., shear forces) during cell dissociation to dislodge cells from microcarriers, which is currently the industry standard. This unit operation must be done quickly and efficiently, as over-exposure to trypsin and long-term exposure to high shear forces can be harmful to the cells. To address some of limitations of conventional harvest enzymes, new versions of dissociation reagents have been developed with modified enzymes that are gentler and safer for cells, as well as animal component-free materials, made in cGMP-compliant facilities and in a ready-to-use solution which will help to ease future regulatory burden. After the dissociation enzymes have performed their function of detaching the cells from the microcarriers, quenching the activity of the enzymes is critical for maintaining viability of the bulk cell solution for further downstream processing. For quenching the enzyme, 10% fetal bovine serum in phosphate buffered saline, complete media, or spent media have been used, but the presence of animal-derived components and variation in efficacy of this quench solution pose challenges to its use in robust, reproducible, cGMP-aligned manufacturing processes. Thus, there is a definitive need to develop a xeno-free alternative with validated inactivation of dissociation enzymes to ensure optimal bioprocessing conditions.

Harvesting of cells from microcarriers is then immediately followed by filtration of the cell/microcarrier slurry through a porous mesh that traps the microcarriers, while cells flow through. Confirming complete removal of the microcarriers from the cell therapy product is absolutely critical to ensuring safety for the patient. At the small (0.1L to 5L) to medium (5L to 20L) scale bioreactor size, conventional flow filtration technology has worked, but when the scale increases to 50L and more, the volume of product to process can overwhelm most systems. Upscaled technologies, like continuous flow centrifugation (kSep), have been tested for cell suspensions, but will require development and validation for each specific process (40). Finally, cell formulation, filling and finish, being the most critical unit operation of MSC production, due to their time-sensitivity, will need to be automated to generate thousands of product vials per batch. A new and promising technology innovation is the development of completely dissolvable microcarriers which has the potential to obviate the cell/microcarrier separation unit operation in the future, markedly streamlining and simplifying the downstream processing of MSCs expanded in microcarrier-based bioreactor systems (41). As the downstream processing solutions are generated for optimizing cell yield and health upon harvest from large scale bioreactors, costs of MSC manufacturing will drop. This cost reduction will transfer to the scientist in the lab or the physician in the clinic, who can then focus on developing the next great application using MSCs.

### Addressing the pricing of products using MSCs

An additional technology challenge is delivering a product that has been manufacturing with economics that allow for reasonable pricing for the patient. The regulatory and reimbursement strategies employed for product pricing and payment are complex and vary globally, so for this manuscript, the focus is on manufacturing sciences. Improved manufacturing processes will decrease cost of goods and ultimately reduce prices for patients. Similar to microchips, hMSCs have experienced log changes in manufacturing costs and over the last 10 years, hMSC costs have gone from about $1,000 per million cells to about $100 per million MSCs. This should continue and bring the cost per million of hMSCs down to $10 over the next 10 years. For a deeper discussion on cost of goods planning and a guide for economic production of cell therapy products, the reader is directed to work by Lipsitz and collaborators (42). In short, as hMSC manufacturing becomes cheaper with economy of scale, this decrease in production cost should translate into a lower price of the final product that the patient sees. Further, the lower cost of the hMSCs will drive demand and consumption, as hMSC technologies will become democratized and available for widespread adoption into new products and platforms.

### Disruptive technologies—what technologies threaten MSC demand?

MSCs are laying the groundwork in terms of establishing regulatory guidelines with the FDA, which will allow for streamlined integration of other cell therapy products that will ultimately disrupt MSC demand and displace it as a technology. The leading candidates for disrupting MSCs are MSC-EVs, systems and synthetic biology, and induced pluripotent stem cells (IPSCs) (Figure 8). MSC derivatives such as MSC-EVs can effectively utilize the therapeutic potential of stem cells without the cell and have the potential to be functionalized with targeting abilities. Regardless, MSCs will still be required to manufacture the MSC-EVs, so demand would take a hit, but not be displaced completely. Systems and synthetic biology technologies tout the ability to enhance therapeutic targeting, homing, biodistribution, potency, and more (Figure 6). hMSCs represent a great starting material and delivery vehicle for gene editing technologies, given the excellent safety profile of hMSCs in clinical trials. Given these possibilities, it is unlikely systems and synthetic biology will wipe out MSC demand completely, but rather complement it to enhance MSC function. IPSCs are an attractive cell source for therapeutic applications as somatic cells can theoretically be reprogrammed to be in an embryonic-like state, which enables pluripotent differentiation potential and development into any desired tissue type (43). The capability to differentiate into many tissue types gives a wider range of potential applications for IPSCs, when compared to MSCs. Further, IPSCs provide a renewable source of cells from potentially just one donor, effectively eliminating donor dependence and variability. Recently, it was reported that these pluripotent markers could be incorporated without viral integration, giving promise that long-term safety can be achieved (44). As of December 2017, there are 59 registered clinical trials using IPSCs, compared to 837 registered clinical trials using MSCs. IPSCs should have an easier process to obtain approvals because of the ground work laid by MSCs, but IPSCs still require large human studies to establish safety and to understand the fate of the IPSCs. Due to their pluripotent capabilities and potential to eliminate donor-to-donor variability, IPSCs have the best chance to displace MSCs, and are predicted to dethrone MSCs after 2040. Another thought here is that it is quite possible that in 2060 preventative medicine technologies and diagnostics for early detection will be developed to treat many diseases and illness before they even manifest.

## High volume and economically manufactured MSCs are the transistors of the future commercial regenmed field

**Figure 9:**
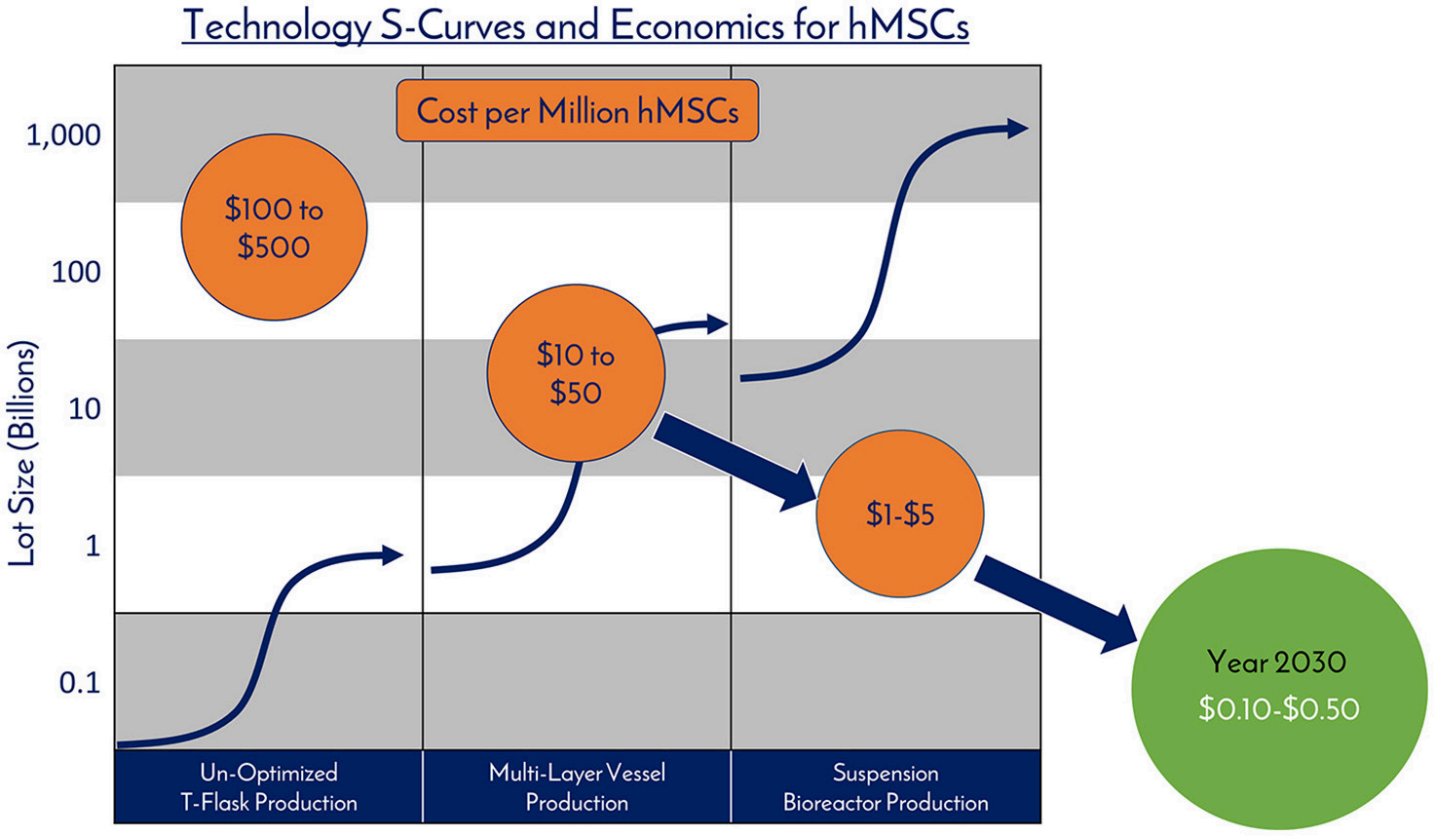
Technology S-Curves and economics for hMSCs. hMSC production processes follow technology development S-Curves as they are adopted and integrated into products and processes. In general, a new hMSC production technology or method is developed, then achieves widespread adoption as a production platform and becomes a standard. This translates to a decrease in the total cost per cell produced due to efficiencies of scale. However, there are diminishing returns as larger scales are required and productivity plateaus until pioneering developments provide a breakthrough another production technology.

While hMSCs are being consumed in large quantities in the development of and performing clinical trials (60 billion per trial), the future commercial demand for hMSCs due to emerging technologies and new markets could cause a sharp increase in demand. The use of hMSCs in clinical trials has caused a mandate for a *consistent* product, shifting the use of hMSCs at variable passage and population doubling levels to cells derived from a consistent process, changing the convention of hMSC usage in academic and preclinical settings to a standardized hMSC product that can be used in clinical settings. At the moment, there is a large supply of hMSCs with high cost, which constricts widespread adoption, but this can be reduced with economies of scale (Figure 9). This highlights the need for process development and manufacturing sciences to keep pace with scalable platform technologies to fulfill clinically and commercially relevant lot sizes. As an intermediate, having access to an hMSC source that has been tested early on for “manufacturability” and mimics a clinical-grade product will help reduce the development timeline to clinic.

**Figure 10:**
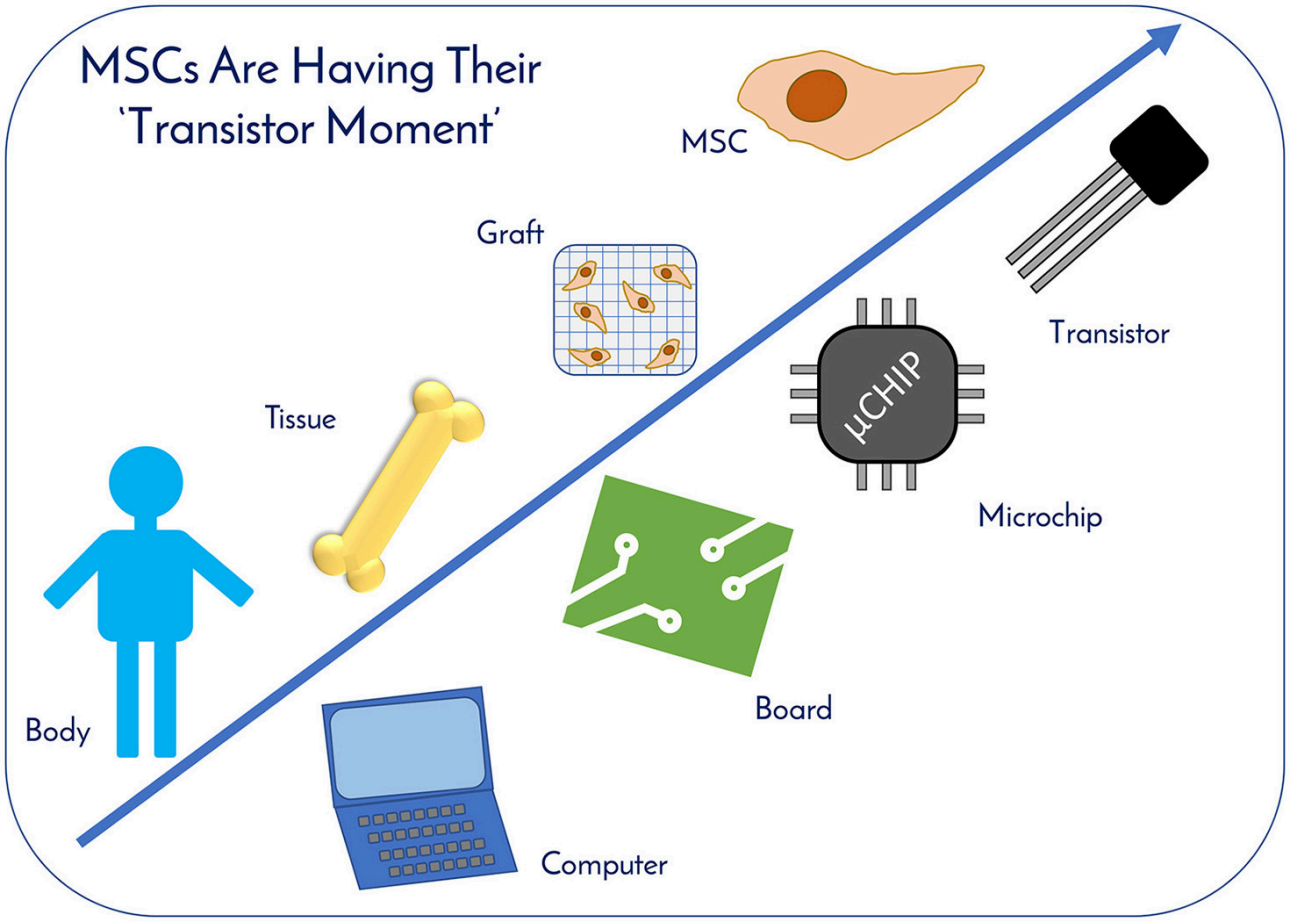
MSCs Are Having Their “Transistor Moment.” hMSCs are no different than technologies like transistors, as they are a highly technical and modular product that requires stringent control over manufacturing that can allow for high quality and consistent performance. MSCs therapeutic applicability is increasing exponentially, drawing parallels to computing. With enhanced economies of scale through improved manufacturing sciences, MSCs are primed for widespread usage as the “microchips” of tomorrow's RegenMed products.

hMSCs are no different than technologies like transistors, as they are a highly technical and modular product that requires stringent control over manufacturing that can allow for high quality and consistent performance (Figure 10). The ability to stack transistors in increasing densities, and at lower costs, on microchips led to the computer revolution as we know it by exponentially increasing computing power, and hMSCs are poised to elicit the same influence on the RegenMed field. hMSC production processes follow technology development S-Curves as they are adopted and integrated into products and processes (Figure 9) (45). In general, a new hMSC production technology or method is developed, then achieves widespread adoption as a production platform and becomes a standard. This translates to a decrease in the total cost per cell produced due to efficiencies of scale. However, there are diminishing returns as larger scales are required and productivity plateaus until pioneering developments provide a breakthrough another production technology. Over the years, the hMSC industry has evolved from manufacturing in un-optimized T-flasks, to multi-layer vessel production processes, and now suspension bioreactor systems. With each technology development, lot size capacity increases and manufacturing costs ultimately drop with scaling efficiencies. Here, the metric that we use for technological improvement is “Cost per Million hMSCs,” which is the central metric related to commercial feasibility.

3D microcarrier based bioreactor systems are a strong candidate for fostering the next breakthrough and can scale to >500L batch sizes to generate 100s of billions of cells per manufacturing lot. The technical challenges manifest themselves in media productivity and downstream processing efforts (41). As next generation solutions are developed and optimized, the authors believe that the industry will see the cost per million hMSCs fall from the $10s today to the range of $0.10–$0.50 over the next 10–15 years. We believe that the near future holds a time where hMSCs are an abundant critical raw material, driving widespread adoption into applications, this combination of abundance and adoptions will spur innovations, leading to even more unexpected applications for hMSCs.

MSCs are the single most used cell type in RegenMed and are on the brink of radically changing how the world of medicine operates. Their unique characteristics, potential to treat many indications, and established safety profile in clinical trials have contributed to their current demand and will only fuel future demand. With enhanced economies of scale through improved manufacturing sciences, MSCs are primed for widespread usage as the “microchips” of tomorrow's RegenMed products. Delivering a safe and effective product is key, and effective guidance from organizations like the FDA and the ARM (Alliance for Regenerative Medicine) will ease and accelerate the translation of MSC technologies from the benchtop to bedside. Ultimately, MSCs are poised to have their “transistor” moment and the stage has been set for their wide spread commercial success impacting many industries for years to come.

## Author contributions

TO and KN prepared manuscript figures. TO wrote the manuscript. TO, LL, TA, KN, and JR provided stem cell expertise, industry knowledge, academic knowledge, and developing technologies expertise for guiding the content of the manuscript. LL, TA, and JR edited the manuscript.

### Conflict of interest statement

TO, KN, LL, TA, and JR are employees of RoosterBio Inc. JR is the Chief Technology Officer of RoosterBio Inc. and maintains ownership. RoosterBio Inc. is a human stem cell manufacturing company focused on accelerating the cell-based bioeconomy by providing standardized stem cell product platforms that enable rapid clinical and commercial translations. This paper is the reflection of opinions generated based on industry experience and knowledge in the field. The authors have no affiliations or financial involvement with any organization or entity with a financial interest in or financial conflict with the subject matter or materials discussed in the manuscript. No writing assistance was utilized in the production of this manuscript.

## Supplementary material

Supplemental Figure 1 Estimated hMSC consumption in academic and preclinical research from 1990 to 2016. Here, the PubMed search criteria for determining total articles and hMSC consumption from academic and preclinical work from 1990 to 2016 in cell therapy and tissue engineering fields are detailed.

Supplemental Figure 2 Estimated hMSC consumption in clinical trials from 1995 to 2016. clinicaltrials.gov was utilized to determine the number of registered clinical trials that listed dosage and enrollment information. These trials were used to calculate hMSCs per patient, total cells consumed per trial, and patients per trial.

## References

[1] Squillaro T Peluso G Galderisi U. Clinical trials with mesenchymal stem cells: an update. Cell Transplant. (2016) 25:829–48. 10.3727/096368915x689622 26423725

[2] Nguyen BNB Ko H Moriarty RA Etheridge JM Fisher JP. Dynamic bioreactor culture of high volume engineered bone tissue. Tissue Eng Part A (2016) 22:263–71. 10.1089/ten.tea.2015.0395 26653703 PMC4779290

[3] Maris B Medicine's transistor moment 8 emerging technologies that could revolutionize the life sciences. Medium (2015) Available online at: https://library.gv.com/medicine-s-transistor-moment-fb6c88f4352f

[4] Diamandis PH Kotler S Diamandis PH, Abundance: The Future Is Better Than You Think. (2012). Available online at: http://www.amazon.com/abundance-future-better-than-think/dp/1451614217/ref=sr_1_1?ie=UTF8&qid=1403624640&sr=8-1&keywords=abundance+kotler

[5] Carlson RH Biology is technology: the promise, peril, and new business of engineering life. J Evol Technol. (2010) **304**:288. 10.1001/jama.2010.1552

[6] McPhate M California Today: Waiting on the Promise of Stem Cells. New York Times (2017) Available online at: https://www.nytimes.com/2017/05/04/us/california-today-stem-cell-research.html

[7] Friedenstein AJ Piatetzky-Shapiro II Petrakova K V. Osteogenesis in transplants of bone marrow cells. J Embryol Exp Morphol. (1966) 16:381–390. 5336210

[8] FDA The Drug Development Process. (2015) Available online at: http://www.fda.gov/forpatients/approvals/drugs/default.htm

[9] Ferrara JLM Levine JE Reddy P Holler E. Graft-versus-host disease. Lancet (2009) 373:1550–61. 10.1016/S0140-6736(09)60237-3 19282026 PMC2735047

[10] American Diabetes Association, Statistics About Diabetes: Overall Numbers, Diabetes Prediabetes. (2017) Available online at: http://www.diabetes.org/diabetes-basics/statistics/?loc=superfooter

[11] U.S. Department of Health and Human Services. Organ Donation Statistics. (2017) Available online at: https://www.organdonor.gov/statistics-stories/statistics.html

[12] Ziegler-Graham K MacKenzie EJ Ephraim PL Travison TG Brookmeyer R. Estimating the Prevalence of Limb Loss in the United States: 2005 to 2050. Arch Phys Med Rehabil. (2017) 89:422–9. 10.1016/j.apmr.2007.11.005 18295618

[13] Grand View Research 3D Bioprinting Market Size to Be Worth $1.82 Billion by 2022. (2017). Available online at: http://www.grandviewresearch.com/press-release/global-3d-bioprinting-market

[14] Miller JS. The billion cell construct: will three-dimensional printing get us there? PLoS Biol. (2014) **12**:e1001882. 10.1371/journal.pbio.1001882 24937565 PMC4061004

[15] Clavien PA Petrowsky H DeOliveira ML Graf R. Strategies for safer liver surgery and partial liver transplantation. N Engl J Med. (2007) 356:1545–59. 10.1056/NEJMra065156 17429086

[16] Bianconi E Piovesan A Facchin F Beraudi A Casadei R Frabetti F Vitale L Pelleri MC Tassani S Piva F. An estimation of the number of cells in the human body. Ann Hum Biol. (2013) 40:463–71. 10.3109/03014460.2013.807878 23829164

[17] Chan C Berthiaume F Nath BD Tilles AW Toner M Yarmush ML. Hepatic tissue engineering for adjunct and temporary liver support: critical technologies. Liver Transplant. (2004) 10:1331–42. 10.1002/lt.20229 15497161

[18] Wang B Zhao L Fish M Logan CY Nusse R. Self-renewing diploid Axin2(+) cells fuel homeostatic renewal of the liver. Nature (2015) 524:180–5. 10.1038/nature14863 26245375 PMC4589224

[19] Snykers S De Kock J Tamara V Rogiers V Hepatic Differentiation of mesenchymal stem cells: *in vitro* strategies. In: Vemuri, M. Chase, L. G. Rao M. S. editors. Mesenchymal Stem Cell Assays and Applications. Totowa, NJ: Humana Press, p. 305–314.

[20] Godoy P Hewitt NJ Albrecht U Andersen ME Ansari N Bhattacharya S. Recent advances in 2D and 3D *in vitro* systems using primary hepatocytes, alternative hepatocyte sources and non-parenchymal liver cells and their use in investigating mechanisms of hepatotoxicity, cell signaling and ADME. Arch Toxicol. (2013) 87:1315–530. 10.1007/s00204-013-1078-5 23974980 PMC3753504

[21] Von Bahr L Batsis I Moll G Hägg M Szakos A Sundberg B. Analysis of tissues following mesenchymal stromal cell therapy in humans indicates limited long-term engraftment and no ectopic tissue formation. Stem Cells (2012) 30:1575–8. 10.1002/stem.1118 22553154

[22] Gnecchi M Zhang Z Ni A Dzau VJ. Paracrine mechanisms in adult stem cell signaling and therapy. Circ Res. (2008) 103:1204–19. 10.1161/CIRCRESAHA.108.176826 19028920 PMC2667788

[23] Ng KS Kuncewicz TM Karp JM. Beyond Hit-and-Run: Stem Cells Leave a Lasting Memory. Cell Metab. (2015) 22:541–3. 10.1016/j.cmet.2015.09.019 26445510

[24] Rani S Ryan AE Griffin MD Ritter T. Mesenchymal stem cell-derived extracellular vesicles: toward cell-free therapeutic applications. Mol Ther. (2015) 23:812–23. 10.1038/mt.2015.44 25868399 PMC4427881

[25] Vader P Mol EA Pasterkamp G Schiffelers RM. Extracellular vesicles for drug delivery. Adv Drug Deliv Rev. (2016) 106:148–56. 10.1016/j.addr.2016.02.006 26928656

[26] Yeo RWY Lai RC Zhang B Tan SS Yin Y Teh BJ Lim SK. Mesenchymal stem cell: An efficient mass producer of exosomes for drug delivery. Adv Drug Deliv Rev. (2013) 65:336–41. 10.1016/j.addr.2012.07.001 22780955

[27] Crivelli B Chlapanidas T Perteghella S Lucarelli E Pascucci L Brini AT. Mesenchymal stem/stromal cell extracellular vesicles: from active principle to next generation drug delivery system. J Control Release (2017) 262:104–17. 10.1016/j.jconrel.2017.07.023 28736264

[28] James S Ng KS Mead BE Dopson S Reeve B Edwards J Extracellular vesicles commercial potential as byproducts of cell manufacturing for research and therapeutic use. Bioprocess Int. (2015) 13:20–8.

[29] Kordelas L Rebmann V Ludwig AK Radtke S Ruesing J Doeppner TR. MSC-derived exosomes: A novel tool to treat therapy-refractory graft-versus-host disease. Leukemia (2014) 28:970–3. 10.1038/leu.2014.41 24445866

[30] Willis GR Fernandez-Gonzalez A Anastas J Vitali SH Liu X Ericsson M Mesenchymal stromal cell exosomes ameliorate experimental bronchopulmonary dysplasia and restore lung function through macrophage immunomodulation. Am J Respir Crit Care Med. (2017) 44:1–68. 10.1164/rccm.201705-0925OC PMC5765387 28853608

[31] Liang X Ding Y Zhang Y Tse HF Lian Q. Paracrine mechanisms of mesenchymal stem cell-based therapy: current status and perspectives. Cell Transplant. (2013) 23:1–32. 10.3727/096368913X667709 23676629

[32] Parekkadan B Van Poll D Suganuma K Carter EA Berthiaume F Tilles AW. Mesenchymal stem cell-derived molecules reverse fulminant hepatic failure. PLoS ONE (2007) **2**:e941. 10.1371/journal.pone.0000941 17895982 PMC1978513

[33] Albersen M Fandel TM Lin G Wang G Banie L Lin CS. Injections of adipose tissue-derived stem cells and stem cell lysate improve recovery of erectile function in a rat model of cavernous nerve injury. J Sex Med. (2010) 7:3331–40. 10.1111/j.1743-6109.2010.01875.x 20561166 PMC3885341

[34] Wood L Global Cosmeceuticals Market Worth USD 61 Billion by 2020 - Analysis, Technologies & Forecasts Report 2016-2020 - Key Vendors: Avon, Bayer, Johnson & Johnson - Research and Markets. Bus Wire (2016) Available online at: https://www.businesswire.com/news/home/20160323006461/en/global-cosmeceuticals-market-worth-usd-61-billion

[35] van der Weele C Tramper J. Cultured meat: Every village its own factory? Trends Biotechnol. (2014) 32:294–6. 10.1016/j.tibtech.2014.04.009 24856100

[36] Post MJ. An alternative animal protein source: cultured beef. Ann N Y Acad Sci. (2014) 1328:29–33. 10.1111/nyas.12569 25376889

[37] Marks L. The birth pangs of monoclonal antibody therapeutics: the failure and legacy of centoxin. MAbs (2012) 4:403–12. 10.4161/mabs.19909 22531443 PMC3355486

[38] Ecker DM Jones SD Levine HL. The therapeutic monoclonal antibody market. MAbs (2015) 7:9–14. 10.4161/19420862.2015.989042 25529996 PMC4622599

[39] Rowley J Abraham E Campbell A Brandwein H Oh S Meeting lot-size challenges of manufacturing adherent cells for therapy. Bioprocess Int. (2012) 10:16–22.

[40] Pattasseril J Varadaraju H Lock L Rowley JA Downstream technology landscape for large-scale therapeutic cell processing. Bioprocess Int. (2013) 11:38–47.

[41] Olsen TR Lock LT Rowley JA Scaling up: how manufacturing sciences will dictate the future of cell therapy. Regen Med. (2016) 11:S15–8. 10.2217/rme-2016-1108s3

[42] Lipsitz YY Milligan WD Fitzpatrick I Stalmeijer E Farid SS Tan KY. A roadmap for cost-of-goods planning to guide economic production of cell therapy products. Cytotherapy (2018) 19:1383–91. 10.1016/j.jcyt.2017.06.009 28935190

[43] Yu J Vodyanik MA Smuga-Otto K Antosiewicz-Bourget J Frane JL Tian S. Induced pluripotent stem cell lines derived from human somatic cells. Science (2007) 318:1917–20. 10.1126/science.1151526 18029452

[44] Junying Y Kejin H Kim SO Shulan T Stewart R Slukvin II. Human induced pluripotent stem cells free of vector and transgene sequences. Science (2009) 324:797–801. 10.1126/science.1172482 19325077 PMC2758053

[45] Simaria AS Hassan S Varadaraju H Rowley J Warren K Vanek P. Allogeneic cell therapy bioprocess economics and optimization: single-use cell expansion technologies. Biotechnol Bioeng. (2014) 111:69–83. 10.1002/bit.25008 23893544 PMC4065358

