# Supplementary figures and images for: Peak MSC—Are We There Yet?
Source: Front Med (Lausanne). 2018 Jun 21;5:178. doi: 10.3389/fmed.2018.00178 (PMC6021509; doi:10.3389/fmed.2018.00178)

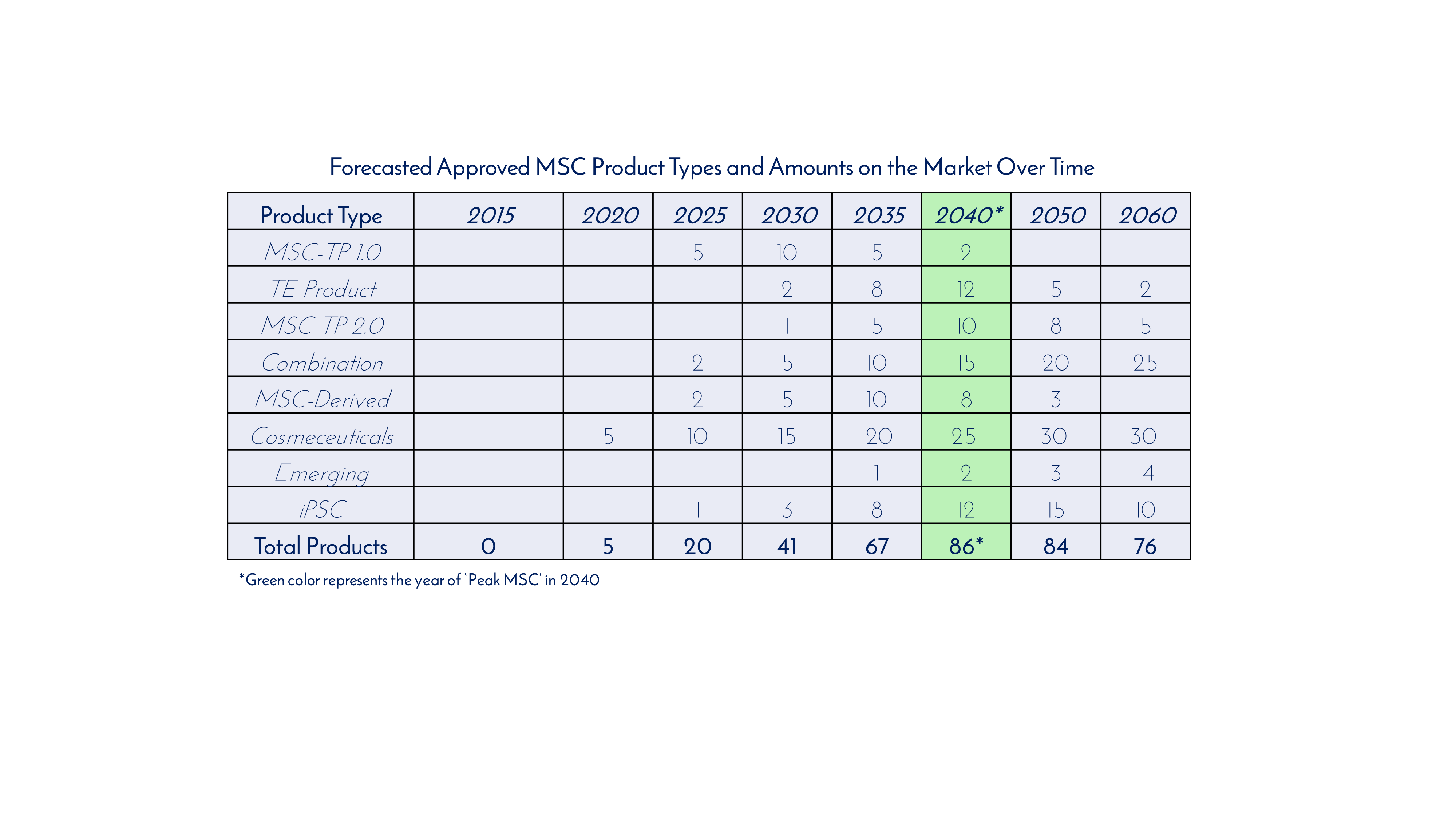

Supplement: Supplemental Figure 1 — Estimated hMSC consumption in academic and preclinical research from 1990 to 2016. Here, the PubMed search criteria for determining total articles and hMSC consumption from academic and preclinical work from 1990 to 2016 in cell therapy and tissue engineering fields are detailed. [file Image_1.TIF]

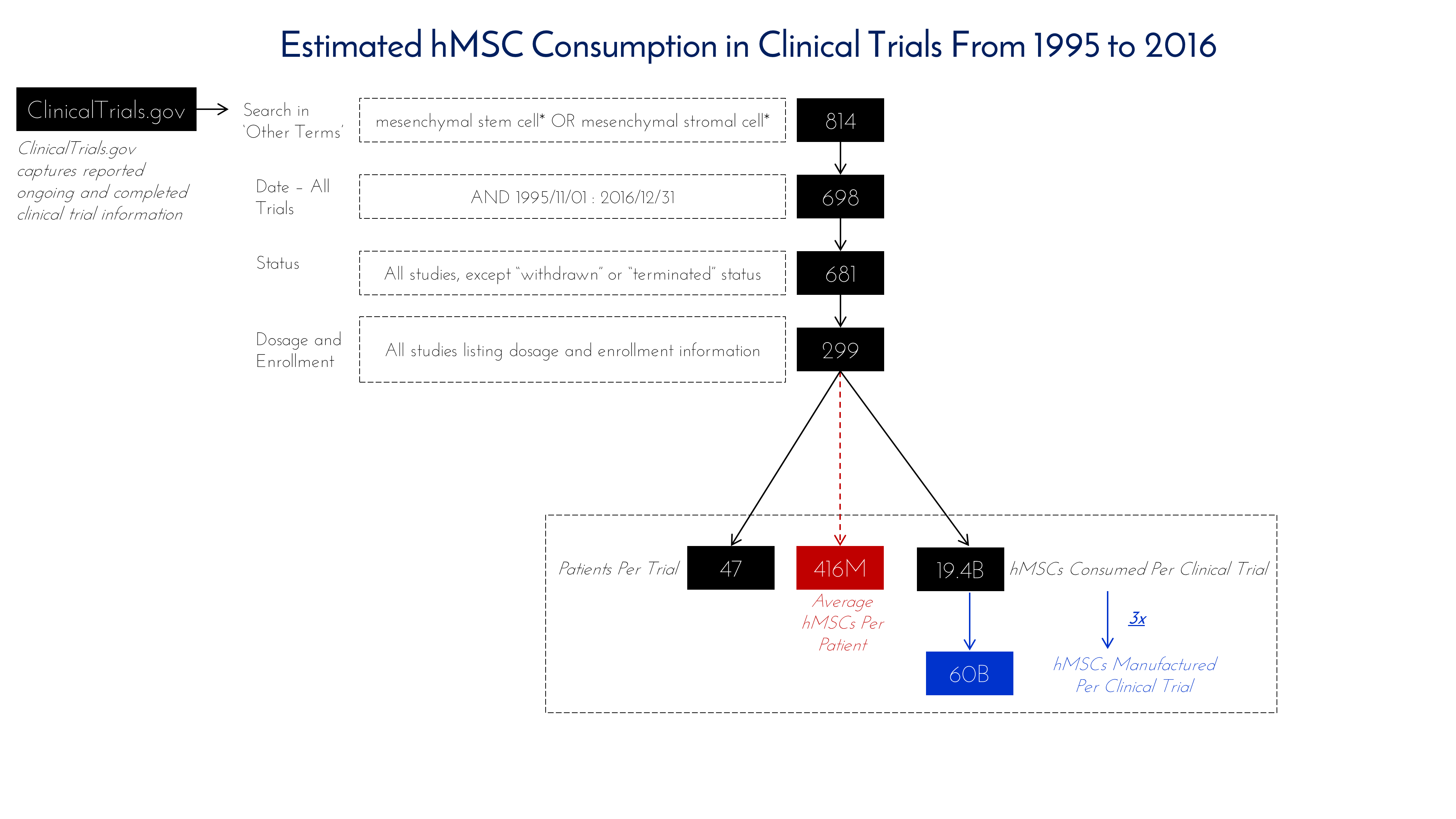

Supplement: Supplemental Figure 2 — Estimated hMSC consumption in clinical trials from 1995 to 2016. clinicaltrials.gov was utilized to determine the number of registered clinical trials that listed dosage and enrollment information. These trials were used to calculate hMSCs per patient, total cells consumed per trial, and patients per trial. [file Image_2.tif]
